# Relationship between parenting measures and parents and child psychopathological symptoms: a cross-sectional study

**DOI:** 10.1186/s12888-020-02778-8

**Published:** 2020-07-17

**Authors:** Monica Bellina, Silvia Grazioli, Marco Garzitto, Maddalena Mauri, Eleonora Rosi, Massimo Molteni, Paolo Brambilla, Maria Nobile

**Affiliations:** 1 Scientific Institute, IRCCS E. Medea, Developmental Psychopathology Unit, Bosisio Parini, Lecco, Italy; 2 Scientific Institute, IRCCS E. Medea, San Vito al Tagliamento, Pordenone, Italy; 3 School of Medicine and Surgery, University of Milano-Bicocca, Milan, Italy; 4 Department of Neurosciences and Mental Health, Fondazione IRCCS Ca’ Granda Ospedale Maggiore Policlinico, University of Milan, Milan, Italy

**Keywords:** Parenting behaviours, Family functioning, Developmental psychopathology, Internalizing and externalizing disorders, Rehabilitation, Treatment, Risk factors, Mediation analysis, Child behavior checklist, Family life questionnaire

## Abstract

**Background:**

Increasing evidence suggests a complex role of family influences, such as the exposure to parent psychopathology through parenting behavior, in parent-to-child psychopathology transmission. Parenting behaviour could represent a relevant target of psychoeducative intervention. Given these premises, we aimed to evaluate homotypic and heterotypic relationships between parent and child psychopathology, mediated by parenting behaviours, taking into account the constructs of parent and offspring internalizing and externalizing psychopathology.

**Methods:**

Internalizing and externalizing symptoms in 272 clinically-referred subjects (mean age = 14.5 ± 2.3; F = 23.5%) and their parents (mothers *n* = 272, fathers *n* = 242) were assessed through the Child Behavior Checklist and the Adult Self Report; four areas of parenting behaviours were investigated through the Family Life Questionnaire. Multiple mediation models were built, considering mother and father psychopathology scales as independent variables, parenting measures and family functioning as mediators (Affirmation, Rules, Discipline and Special Allowances), child psychopathology scales as dependent variables and demographic variables as covariates.

**Results:**

Regression models showed a significant effect of maternal internalizing symptomatology on child externalizing behavioral problems; high levels of maternal pathology predicted high levels of children’s psychopathology. A total mediating effect of parenting measures was found: high levels of internalizing symptoms in mothers predicted low levels of affirmation, which in turn predicted high levels of externalizing psychopathology in children.

**Conclusions:**

Our study results confirmed the existence of interdependent links between mothers’ psychiatric symptomatology, parenting behaviour and offspring outcomes, specifically in an Italian context. On a clinical and rehabilitation basis, this work offers suggestions about parenting practices, specifically maternal, involved in the maintenance of child psychopathology.

## Background

Psychopathological disorders in children and adolescents are quite common. According to national [1] and international [2] epidemiological data, the worldwide prevalence of mental disorders ranges from approximately 6.7% [3] to 13.4% [2, 4]. Concerning the Italian context, DSM-IV disorders among developmental-aged patients is around 8.2%, with a more frequent presence of internalizing disorders (with prominent depressive, anxiety and somatic symptoms; 6.5% of cases) than externalizing disorders (with conspicuous disruptive, impulsive and substance use symptoms; 1.2% of cases). Psychopathology is often stable through time if not detected and addressed early; such evidence highlights the need for early clinical support among children with frailties to reduce the probability of adverse psychopathology outcomes [5].

Theoretical and empirical models suggest a multifactorial aetiology of child psychopathology. International data come from the World Health Organization ‘World Mental Health Surveys’: McLaughlin and colleagues [6] showed that parents’ psychopathology is highly correlated with psychiatric disorder risk in offspring. Exposure to parents’ psychopathology has a crucial role in the development of child psychiatric disorders [6, 7], given the fact that it represents both a genetic and an environmental risk factor.

Together with parents’ psychological functioning, additional factors involved in the development of children’s emotional and behavioural regulation are parenting practices and family functioning [8–10]. The construct of parenting practices involves cognition, emotions and affects daily directed towards children as well as goal-directed behaviors such as showing children support, monitoring, nurturing, encouragement and companionship or putting in place intrusive and punitive behaviours [11]. Positive practices consist of parental warmth, support, positive affect and sensitivity and it is associated with greater levels of self-regulation in children [12], whereas negative parenting comprises punitions, hostility, neglect, excessive intrusiveness and over-control [13] and it is inversely correlated with self-regulation in children [12, 14].

The construct of family functioning can be defined as the capacity of the family system to meet the needs of its members through various behaviours [15]. This dimension has a key role in the psychosocial well-being of all family members and it could represent a therapeutic target in clinical practice [16].

Recent research on parents’ psychological functioning, parenting practices and family functioning highlights the existence of strong relationships between these two areas and offspring psychopathology; in fact, there is a probable mediating effect of parent-child relationship between parent and children’s psychological problems. Aunola and colleagues [17] found a full mediation effect of psychological control (conceived as a parenting dimension) between parents’ depressive symptoms and offspring distress, conceptualized as negative daily emotions. Furthermore, Grasso and colleagues [18], starting from an examination of harsh parenting mediation on the partner violence and disruptive behaviour in children relationship, found a partial mediation by psychological aggression towards the child. Similarly, Lieb and colleagues [19], through a longitudinal study, found an association between internalizing pathology such as social phobia in children and parental overprotection and rejection; these peculiar parenting domains were also associated with parental internalizing symptoms as anxiety disorder. As opposite, Vostanis and colleagues [20] found associations between absence of child psychopathology and rewarding and non-punitive parenting behaviours.

These studies highlighted the existence of strong links between parenting, family functioning and parent and children’s psychopathology. However, the considered works explored single aspects (internalizing or externalizing) of parents’ symptoms and/or children’s psychopathology. Indeed, the literature lacks an overall view of the above-mentioned effects, considering internalizing and externalizing psychopathology in a single integrated model.

Surprisingly, to date, there is a lack of studies addressing this aim in an Italian setting, despite cultural and contextual influences on parenting practices having been previously described [13, 21, 22].

Given the above-mentioned premises, we hypothesized the existence of a relationship between the three constructs of parent psychopathology, parenting practices or family functioning and offspring psychopathology. Our work aims to reproduce, especially in an Italian sample, results that could confirm the potential role of parenting in mediating the links between parent’s and child’s internalizing and externalizing psychopathology.

A mediation analysis was implemented to investigate the association between parents’ internalizing/externalizing symptoms and parenting practices and children’s internalizing/externalizing symptomatology. Parent and child psychopathology were assessed with the Internalizing and Externalizing scales of the Child Behavior Checklist/6–18 (CBCL/6–18) [23] and Adult Self Report (ASR) [24].

## Methods

The present study is part of the Italian longitudinal *Genesis* Project [25], a multicentre research project involving the Child Psychiatry Unit of the ‘Eugenio Medea’ Scientific Institute in Bosisio Parini (LC), Conegliano Veneto (TV), Pasian di Prato (UD) and San Vito al Tagliamento (PN).

### Sample

Our study involved a sample of children and adolescents referred to clinical centres between 2003 and 2011 for emotional and behavioural problems worthy of clinical attention, such as depressive disorders, generalized anxiety disorder, separation anxiety disorder, attention deficit/hyperactivity disorder and oppositional defiant disorder, diagnosed according to DSM-IV criteria. Parents were asked to complete questionnaires about their parenting practices or family functioning and children’s psychopathological symptoms. Participants were excluded if they had an associated neurologic, genetic, infectious or metabolic disorder, or a seizure disorder, cognitive disability (IQ < 70), pervasive developmental disorders, severe hypoacusia, hypovision or severe linguistic comprehension deficit.

The study sample consisted of 272 children and adolescents aged 9–18 and their biological parents (aged 30–65), who completed the evaluation. Specifically, 272 mothers and 242 fathers took part in the study; parents from 242 families were married or partners, whereas 30 participating families were characterized by divorced or separated parents. In the second case, only mothers accepted to participate to the present research project. All children and parents were fluent in Italian.

Table 1 shows the children’s and parents’ demographic characteristics.

**Table 1:** Children’s and parents’ demographic characteristics

	Males (*n* = 208)	Females (*n* = 64)	Total sample (*n* = 272)
Child’s Age (M ± Sd)	14.4 ± 2.3	15.2 ± 2.2	14.5 ± 2.3
Family SES (M ± Sd)	52.8 ± 16.7	52.0 ± 17.8	52.6 ± 16.9
Mother’s Age (M ± Sd)	45.8 ± 4.9	46.2 ± 5.0	45.8 ± 5.0
Father’s Age (M ± Sd)	48.5 ± 5.1	48.1 ± 6.1	48.4 ± 5.4

*Legend: Sd* Standard deviation, *SES* Socio-Economic Status

### Measures

#### Socio-demographic information

The sample demographic characteristics were collected together with family socioeconomic status (SES), evaluated through the Four Factor Index of Social Status [26]. The SES value is measured through the subjects’ education level, employment, marital status and sex. The values are grouped in four categories: 0 = very low SES, 10 to 35 = low SES, 40 to 65 = medium SES and more than 70 = high SES.

#### Parent psychopathology

The ASR [24] is a 126-item self-report questionnaire for adults assessing emotional and behavioural functioning aspects. Both mothers and fathers completed the form, referring to themselves. The questionnaire provides scores for the following syndrome scales: anxious/depressed, withdrawn, somatic complaints, thought problems, attention problems, aggressive behaviour, rule-breaking behaviour and intrusive behaviour. In addition to the syndrome scale, the ASR problem items can be scored in two broad groupings of syndromes. One grouping, designated as Internalizing, consists of the sum of three syndrome scales: anxious/depressed, withdrawn and somatic complaints. This grouping is defined as Internalizing because it comprises problems that exist mainly within the self. The second grouping, designated as Externalizing, consists of the sum of three other syndromes scales: aggressive behaviour, rule-breaking behaviour and intrusive behaviour. The problems comprising the Externalizing grouping mainly involve conflicts with other people and with social mores.

Lastly, the questionnaire provides scores for DSM-oriented scales; however, those scores were not included in our analyses and results since categorical constructs were not the focus of our interest.

Items are rated on a 3-point scale: 0 = Not True, 1 = Somewhat or Sometimes True, 2 = Very True or Often True.

In this study, we took into account the T-scores of the mother Internalizing (M-Int) and Externalizing (M-Ext) Scales, as well as father Internalizing (F-Int) and Externalizing (F-Ext) values. T-scores are standardized values, displayed in a normal distribution with a mean of 50 and a standard deviation of 10 in absolute values.

Psychometric and cross-cultural properties of ASR are given in the relative manual and by Ivanova and colleagues [24, 27].

#### Parenting practices

Parenting practices and family functioning have been investigated with the ‘background section’ (Family Life Questionnaire, FaLQ) of the parent and adolescent versions of the Development and Well-Being Assessment (DAWBA) diagnostic interview [28]. The FaLQ enables the collection of information about the child’s and his or her family’s life context and parenting practices [29] through a 13-item questionnaire investigating four theoretical scales: Affirmation (four items), Rules (two items), Discipline (four items) and Special Allowances (three items). Participants are asked to indicate how well the descriptions in the questionnaire apply to their child using 4-point scale: 0 = Not at all, 1 = A little, 2 = A medium amount, 3 = A great deal. It differs from other parenting assessments by measuring the experience of family/parents in relation to a single child and it allows to assess differences in parent–child and sibling–sibling relationships.

Affirmation is defined as behaviours that the parent puts in place to support or help children in various situations or to show them approval and affection and refers to parent-child relationship; examples of Affirmation items are ‘gets love and affection’, ‘is praised and rewarded’, ‘gets help and support when stressed’ and ‘is liked and respected for who they are’. Affirmation subscale has a large convergent validity (*r* = .56, *p* < .001) with Alabama Positive Parenting subscale [APQ, 30], one of the most used questionnaire concerning parenting practices.

The Rules scale is defined as the ability to create coherent and shared family rules and to enforce them and it measures structure and organization within the family. Examples of Rules items are ‘there are clear rules about what they are expected to do and what they are not allowed to do’ and ‘these family rules are applied consistently’. Last and colleagues [16] found that the divergent validity between the FaLQ Rules and the Alabama inconsistent discipline scale [30] was moderate (*r* = −.45, *p* < .001).

Discipline is defined as behaviours by parents in response to and intended to correct misbehaviour by the children and it refers to punishment; examples of discipline items are ‘told off or corrected for things they do wrong’, ‘physically punished (e.g. a smack or a slap)’ and ‘punished in other ways (e.g. things they like are taken away, grounded, time out)’.

Special Allowances refers to various overprotection behaviours as opposed to lack of supervision; hence, it is related to over- and under-involvement from parents. Examples of allowance items are ‘leads a very protected life’ and ‘spends time by themselves (e.g. with TV, music, games, books)’.

Regarding psychometric properties, Last and colleagues [16] found good internal consistency and test-retest reliability of Affirmation and Rules scales. Discipline subscale had a poor internal consistency but good test-retest reliability, whereas Special Allowance scale had poor internal consistency and moderate test-retest reliability.

#### Children’s psychopathology

The assessment was conducted using the Italian version of the CBCL/6–18 [23, 31], an empirically based checklist of social competence and behavioural problems filled out by participants’ mothers. The questionnaire includes 138 items: 20 items for children’s and adolescents’ social competence and 118 for behavioural and emotional problems. Like the ASR, the CBCL/6–18 is divided into two major factors: the Internalizing scale (with the anxious/depressed, withdrawn and somatic complaints subscales) and the Externalizing scale, which consists of 35 items and two subscales: rule breaking and aggressive behaviour. The consistency between CBCL empirical/dimensional diagnoses and categorical/qualitative ones has been widely empirically evaluated [32].

In this study, we used the T-score of Internalizing (C-Int) and Externalizing (C-Ext) scales based on the Italian population [23], with a normal distribution (mean = 50, standard deviation = 10).

Good internal consistency for Italian versions of the CBCL/6–18 (α > .78 for Total Problems and the two broadband scales, and α > .65 for most narrow band scales) was reported [31].

### Data analyses

#### Preliminary analyses

To describe the sample of children and their parents, frequencies of CBCL and ASR Internalizing and Externalizing Problems scale scores in the clinical range were computed. Based on the ASEBA Multicultural Manual, we considered a clinical range of scores with corresponding T > 63 for the Internalizing and Externalizing Problems scales. Ratings of child and parental psychopathology were computed to describe the sample.

Descriptive analyses were conducted on all of our variables of interest.

We analysed distribution of variables using absolute cut-offs of skewness, kurtosis and distribution plots.

#### Correlation analyses

Correlation analyses were conducted to examine a possible linear relationships between M-Int, M-Ext, F-Int, F-Ext, C-Int, C-Ext and parenting practices. Association between psychopathology, parenting practices and demographic variables of interest was also analysed.

#### Multiple mediation models

Multiple mediation models were built to assess whether the relationship between parents’ and children’s psychopathology severity was mediated by the quality of parenting practices. We hypothesized that the relationship between parents’ psychopathology (M-Int, M-Ext, F-Int, F-Ext) and children’s psychopathology (C-Int, C-Ext) could be partly explained by the mediation of parenting practices (Affirmation, Special Allowances, Discipline and Rules).

We performed these analyses using Jamovi software, version 1.0.7.0, implemented in R [33, 34]. The amplitude of the effects was estimated through bootstrapping methodology with a 1000-resampling iterations process to produce robust bootstrapped standard errors (SEs) and 95% confidence intervals (CIs).

Demographic variables (gender, SES, and parents’ and children’s age) were inserted into the models as covariates to control for confounding variables.

**Fig. 1:**
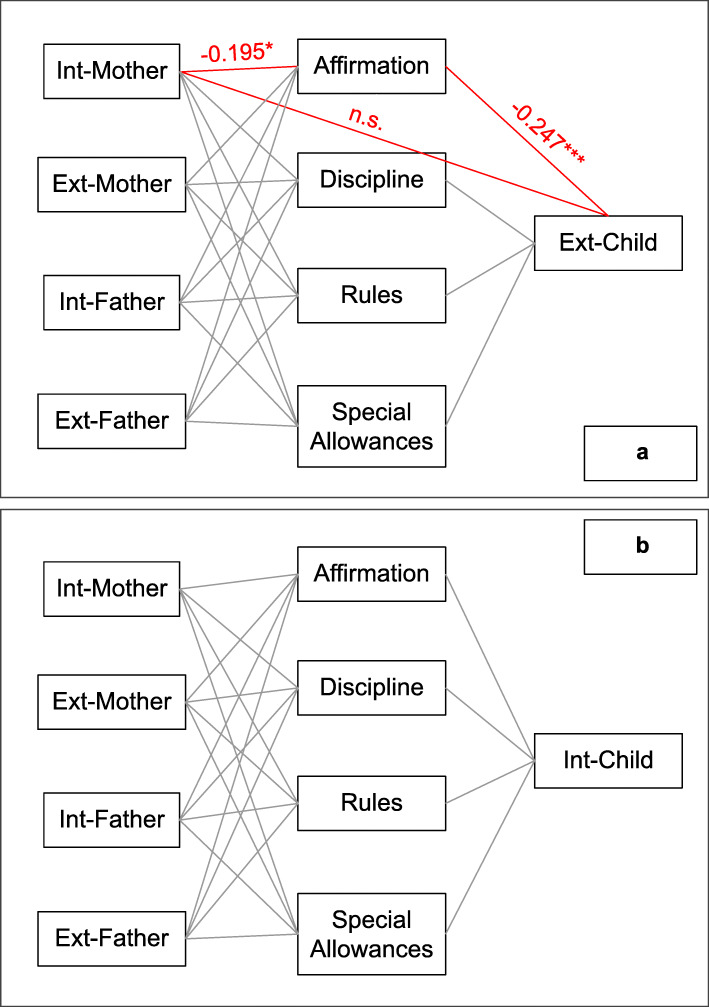
Red line with asterisk: significant parameter; ns: non-significant; *: *p* < 0.05; ***: *p* < 0.001

Two models were built (see Fig. 1).

Model 1: M-Int, M-Ext, F-Int, F-Ext as predictors (independent variables); Affirmation, Special Allowances, Discipline and Rules (parenting practices) as mediators; and C-Ext as outcome variable (dependent variable).

Model 2: M-Int, M-Ext, F-Int, F-Ext as predictors (independent variables); Affirmation, Special Allowances, Discipline and Rules (parenting practices) as mediators; and C-Int as outcome variable (dependent variable).

The total effect explains the relationship between the considered independent variable (M-Int, M-Ext, F-Int or F-Ext, respectively) and the dependent variable (C-Int or C-Ext, respectively) through a simple regression analysis. In this step, values are not partialized out of mediators’ effects.

The direct effect explains the association between the considered independent variable (M-Int, M-Ext, F-Int or F-Ext, respectively) on the dependent variable (C-Int or C-Ext, separately) while keeping the other independent variables and mediator values constant. This effect offers an index of association between a parent psychopathology scale and a child psychopathology scale net of sociodemographic, other parent psychopathology variables and mediators’ effect.

The indirect effect is a value of the association that passes through the mediator, net of the direct effect between parent and child psychopathology. If the indirect effect is significant, a mediation effect occurs.

Full mediation occurs when direct effect is not significant but single regressions between parent psychopathology and parenting measures, firstly, and parenting measures with children psychopathology, secondly, are significant. Partial mediation occurs when the mediator accounts for some, but not all, of the relationships between the independent variable and the outcome. Finally, mediation is rejected when either one of the two regressions between independent variable, mediator and dependent variable is not significant.

## Results

### Preliminary analyses

Table 2 provides an overview of frequencies of Internalizing and Externalizing scores in the clinical range assessed by CBCL and ASR.

**Table 2:** Children’s and parents’ psychopathology measures

	**Internalizing T-values**		**Externalizing T-values**	
	**(M ± Sd)**	**N in clinical range (%)**	**(M ± Sd)**	**N in clinical range (%)**
Mothers (*n* = 272)	55.2 ± 9.6	86 (31.6%)	50.3 ± 8.8	42 (15.4%)
Fathers (*n* = 242)	53.4 ± 9.5	62 (25.7%)	50.4 ± 8.8	40 (16.5%)
Children (*n* = 272)	57.0 ± 9.6	101 (37.1%)	53.4 ± 9.0	67 (24.6%)
	**Categorical diagnosis**			
Children (*n* = 272)	**Type**	**N (%)**		
	Any Behaviour Disorder	15 (5.5%)		
	Any Mood Disorder	35 (12.9%)		
	Any Anxiety Disorder	121 (44.5%)		
	Attention Deficit Hyperactivity Disorder	83 (30.5%)		
	Other Diagnoses	18 (6.6%)		

*Legend: Sd* Standard deviation; Behaviour Disorders (Oppositional Defiant Disorder, Conduct Disorder and Disruptive Disorder NOS), Mood Disorders (Depressive Disorder, Dysthymic Disorder and Depressive Disorder NOS), Anxiety Disorders (Generalized Anxiety Disorder, Specific Phobia, Panic Disorder, Social Phobia, Separation Anxiety, Obsessive–Compulsive Disorder, Post-Traumatic Stress Disorder, Mixed Anxiety Depressive Disorder and other Anxiety Disorders NOS) and Other Diagnoses (all diagnostic conditions that are not an emotional or a behavioural disorder, such as Tic Disorder, Stuttering, Enuresis, and Selective Mutism)

All variables were normally distributed. Distribution plots indicated no extreme outliers for any of the variables of interest.

### Linear correlation analyses

Correlation results are reported in Table 3. Pearson linear correlations indicate that both parents’ internalizing and externalizing problems are positively related to child psychopathology for internalizing and externalizing problems. Regarding parental measures, Affirmation and Rules scales are negatively related to C-Int and C-Ext scales and to M-Int and M-Ext scales; regarding F-Int, F-Ext scales, this result is significant only for the Affirmation subscale.

**Table 3:** Pearson linear correlation coefficients

	C-Ext	C-Int	M-Ext	M-Int	F-Ext	F-Int	Aff.	Rules	Disc.	Spec. All.	SES
C-Int	.477^***^										
M-Ext	.377^***^	.423^***^									
M-Int	.348^***^	.441^***^	.627^***^								
F-Ext	.209^**^	.185^**^	.183^**^	.206^**^							
F-Int	.239^***^	.339^***^	.257^***^	.294^***^	.630^***^						
Aff.	−.359^***^	−.190^**^	−.228^***^	−.293^***^	−.210^**^	−.164^*^					
Rules	−.214^***^	−.213^***^	−.223^***^	−.236^***^	−.081	−.046	.452^***^				
Disc.	.205^*^	.024	.043	−.044	.003	.008	.067	.139^*^			
Spec. All.	.065	−.167^**^	.098	.099	−.009	.099	.040	.076	.179**		
SES	−.106	−.076	−.020	−.108	−.019	.000	.041	.078	−.114	.097	
Age	−.102	−.015	−.076	−.066	.048	−.003	−.039	−.070	−.225^***^	−.002	−.004

*Legend: C-Int* Children’s Internalizing Problem scale, *C-Ext* Children’s Externalizing Problem scale, *M-Int* Mother’s Internalizing Problem scale, *M-Ext* Mother’s Externalizing Problem scale, *F-Int* Father’s Internalizing Problem scale, *F-Ext* Father’s Externalizing Problem scale, *Aff.* Affirmation, *Disc.* Discipline, *Spec. All.* Special Allowances, *Gend.* Gender

*p < .05; **p < .01; ***p < .001

The parenting Discipline scale is positively related only to C-Ext; Special Allowances in parenting is negatively related only to C-Int.

### Multiple mediation models

The model results are depicted in Fig. 1 and Tables 4, 5 and 6. Results from the mediation analysis indicated that M-Int is related to C-Ext through an indirect effect of Affirmation. The occurring relationship was direct: high levels of mother’s psychopathology corresponded to high levels of child’s symptomatology. As shown in Table 6, the total effect was significant (*p* = 0.045), with a β coefficient of 0.155 (also confirmed by bootstrap percentile CI).

**Table 4:** Mediation model, indirect (mediated) effects

Effect			β coefficient (Bootstrap percentile CI)	***SE***	z	***p***
I.V.	Mediator	D.V.				
Mother - Internalizing	Affirmation	Child - Externalizing	**0.048 (0.009; 0.095)**	0.022	2.052	**0.040**
	Discipline		− 0.035 (− 0.083; 0.008)	0.023	−1.428	0.153
	Rules		0.010 (−0.008; 0.037)	0.012	0.824	0.410
	Special Allow.		0.002 (− 0.010; 0.017)	0.006	0.359	0.719
Mother - Externalizing	Affirmation		0.022 (− 0.018; 0.065)	0.021	1.134	0.257
	Discipline		0.021 (−0.028; 0.074)	0.025	0.861	0.389
	Rules		0.020 (−0.004; 0.058)	0.017	1.241	0.215
	Special Allow.		−0.001 (− 0.012; 0.011)	0.006	− 0.154	0.878
Father - Internalizing	Affirmation		−0.009 (− 0.054; 0.036)	0.022	− 0.381	0.703
	Discipline		−0.004 (− 0.037; 0.038)	0.019	− 0.180	0.857
	Rules		−0.009 (− 0.035; 0.008)	0.011	− 0.768	0.442
	Special Allow.		0.004 (−0.012; 0.033)	0.011	0.390	0.697
Father - Externalizing	Affirmation		0.044 (−0.003; 0.101)	0.026	1.777	0.076
	Discipline		0.006 (−0.035; 0.043)	0.020	0.291	0.771
	Rule		0.012 (−0.007; 0.045)	0.013	0.993	0.321
	Special Allow.		−0.004 (− 0.027; 0.011)	0.009	− 0.418	0.676

Parenting style mediating parents’ internalizing and externalizing psychopathology (I.V.) and child’s externalizing psychopathology (D.V.). Covariates: gender, SES, children’s age and parent’s age

**Table 5:** Mediation model, indirect (mediated) effects

Effect			β coefficient (CI)	***SE***	z	***p***
I.V.	Mediator	D.V.				
Mother - Internalizing	Affirmation	Child - Internalizing	−0.009 (− 0.044; 0.018)	0.014	− 0.584	0.560
	Discipline		0.002 (−0.015; 0.025)	0.010	0.191	0.849
	Rules		0.015 (−0.014; 0.047)	0.015	0.946	0.344
	Special Allow.		0.011 (−0.010; 0.039)	0.012	0.920	0.358
Mother - Externalizing	Affirmation		−0.004 (− 0.032; 0.008)	0.010	− 0.428	0.669
	Discipline		−0.001 (− 0.022; 0.013)	0.008	− 0.146	0.884
	Rules		0.028 (0.001; 0.085)	0.021	1.454	0.146
	Special Allow.		−0.004 (−0.029; 0.023)	0.012	−0.335	0.738
Father - Internalizing	Affirmation		0.002 (−0.012; 0.021)	0.008	0.200	0.841
	Discipline		0.001 (−0.010; 0.011)	0.005	0.041	0.967
	Rules		−0.012 (− 0.045; 0.0140)	0.014	− 0.859	0.390
	Special Allow.		0.021 (−0.020; 0.056)	0.015	1.412	0.158
Father - Externalizing	Affirmation		−0.008 (− 0.050; 0.016)	0.015	− 0.557	0.578
	Discipline		−0.001 (− 0.013; 0.010)	0.005	− 0.061	0.951
	Rules		0.017 (−0.010; 0.057)	0.016	1.156	0.248
	Special Allow.		−0.018 (− 0.052; 0.001)	0.014	−1.399	0.162

Parenting style mediating parents’ internalizing and externalizing psychopathology (I.V.) and child’s internalizing psychopathology (D.V.). Covariates: gender, SES, children’s age and parent’s age. I.V.: Independent Variable; *D.V.* Dependent Variable, *SES* Socio-Economic Status, *Special Allow.* Special Allowances, *CI* confidence interval, *SE* Standard Error, *z* Goodman Test value, *p* probability value

**Table 6:** Focus on the significant mediating effect of parenting style: Affirmation

Effect	β coefficient (Bootstrap percentile CI)	***SE***	z	***P***
Direct				
Int-Mother ➔ Ext-Child	0.130 (−0.002; 0.256)	0.066	1.823	0.068
Component				
Int-Mother ➔ Affirmation	**−0.195 (− 0.069; − 0.008)**	0.015	−2.394	**0.017**
Affirmation ➔ Ext-Child	**−0.247 (−1.771; − 0.660)**	0.287	−4.260	**< .001**
Total				
Direct + Indirect	**0.155 (0.004; 0.286)**	0.072	2.008	**0.045**

Direct effect, specific indirect effect and total effect of maternal internalizing psychopathology on children’s externalizing psychopathology through Affirmation (parenting style). Covariates: gender, SES, children’s age and parent’s age

*SES* Socio-Economic Status, *Int:* Internalizing, *Ext* Externalizing, *CI* confidence interval, *SE* Standard Error, z Goodman Test value, *p* probability value

Regression results showed a significant (*p* = 0.017) negative effect of M-Int on Affirmation, with a β coefficient of − 0.195; moreover, regression analysis with Affirmation as an independent variable and C-Ext as a dependent variable was significant (*p* < 0.001) with a β coefficient of − 0.247. Specifically, high levels of M-Int were related to low Affirmation, which in turn was associated with high C-Ext.

The direct effect was not significant (*p* = 0.068); thus, the mediating effect of Affirmation between M-Int and C-Ext was total (Table 6).

No other significant mediating effects were found in our multiple mediation models.

## Discussion

Our work focused on disentangling the potential role of parenting practices mediating the complex relationship between parent’s and child’s psychopathology in an Italian sample of children with internalizing and externalizing symptomatology. In fact, to the best of our knowledge, there is a lack of previous literature regarding this topic in an Italian setting, despite cultural and contextual influences on parenting dimensions having been previously described [22]. Furthermore, it is important to note that our findings should be considered relevant for all Italian socioeconomic statuses, given the lack of correlation effect between the SES variable and the clinical data in adults and children.

The association between parental and child psychiatric disorders has been well established by previous researchers [20, 35]. Concerning our study, the results suggested the existence of significant associations between maternal and child psychopathological symptoms through a full mediation of parenting practices, as previous research proposed [17]. Specifically, our mediation models results showed, in an Italian setting, that the association between mothers’ internalizing symptomatology and children’s externalizing psychopathology was fully mediated by Affirmation dimension, a measure of supportive practices and displays of approval and affection by parents towards their offspring. Specifically, we found that general mother’s internalizing psychopathology explains lower levels of affective practices. Those practices could lead, in turn, to an increase in children’s maladaptive and externalizing symptoms, as we hypothesized. On the contrary, our results suggest that positive supportive practices lead to increased functional conduct in the offspring, as highlighted in previous literature [36].

Our study results are partly consistent with Aunola and colleagues’ work, which focused on parental depressive symptomatology, parenting (operationalized as psychological control in daily interaction and parental affection) and children’s distress (parent- and teacher-reported daily negative emotions expressed by the child).

Previous studies concentrated on single categorical diagnoses [19]. Therefore, our findings enlarge the focus and suggest that overall maternal internalizing symptomatology has a link with general child’s externalizing psychopathology. Moreover, our study targeted child’s psychopathology in a clinical sample and not only the distress construct or a single diagnosis, such as antisocial or conduct problems [20, 37].

Unlike other studies, we did not find correlations between Discipline and Special Allowances and parental symptoms. This result may be explained, on one hand, by the effective absence of any link with parental psychopathology. On the other hand, it could be reconducted to a statistical bias effect. Indeed, Last and colleagues [16] preliminarily found that the Discipline scale of the FaLQ showed poor internal consistency but good test–retest reliability; Special Allowances showed poor internal consistency and moderate test-retest reliability. Future investigations could better clarify FaLQ validity and explain the connections with parental psychopathology.

Our study, compared to previous works [17, 19, 20, 37], also took into account father psychopathology. Our results showed that both father’s externalizing and internalizing symptoms are correlated with child psychopathology, confirming recent evidence that underlines the importance of father inclusion and his role in clarifying child symptomatology [38–41]. However, we did not find any effects in the mediation model about father psychopathology and parenting practices. The literature on this topic explains similar findings through the fact that in most families mothers are the primary caregiver, and children may be more influenced by the caregiver who is most involved in their lives [42–44] .Indeed, only a few papers [17, 37], found that the relationship between child and father psychopathology is mediated by overprotection. Further studies would better define the relationship between child’s and father’s psychopathology and father’s practices.

Concerning maternal parenting dimensions, we observed that Affirmation has a relevant role in mediating mother and child symptoms. This could represent an implication to be taken into account in clinical and educational areas.

Although the present work employed a referred clinical sample, it still has some limitations. First, the use of multiple measures obtained by the same informants (i.e. parents) could suffer from shared method variance and inflate the estimates of results. To address this limitation, further studies regarding internalizing and externalizing psychopathology reported by multiple informants are needed. Second, although the results are similar to those of other studies, the participants were recruited from an Italian sample, which potentially limits the results’ replicability in other countries, despite addressing a probable context-dependent issue [22]. Third, because we used a cross-sectional design, our findings provide only a static view of the data: the mediation model’s causal nature could be better addressed in further longitudinal studies. Therefore, our results do not provide information on the onset of, progress of or changes in the symptoms over time. For these reasons, it is not possible to provide any certainty on the direction of effects. Moreover, since parental reports regarding their parenting practices and their family functioning are often biased as parents tend to give socially desirable answers and avoid reporting problematic behaviors to a third party [44, 45], it would be desirable to include observational assessments around parenting functioning. Despite this limitation, FaLQ could be a brief and quick instrument that helps clinicians to understand parenting practices and eventually to program a psychoeducative treatment.

Lastly, a genetic characterization was not included in our work but in future research it could further disentangle environmental versus genetic influences on familial psychopathology transmission. Despite genetic factors being relevant to explain the complex relationships between child and parent psychopathology, our results provide suggestions for health and welfare systems, as well as for professionals in planning preventive programmes in an Italian context.

## Conclusions

Our work confirmed, in an Italian setting, previous evidence of the existence of psychopathology links between members of a family who share environmental and genetic factors. From a clinical perspective, it is overall relevant to note that in a psychiatric healthcare system characterized by poor economic resources, the effect of parental psychopathology on child psychiatric outcomes should not be neglected; effective treatment models would include mother and child in rehabilitation programs for psychiatric and psychological frailties. The mother’s symptomatic improvement through clinical treatments, together with parent training, could be significantly related to the child’s subsequent psychiatric changes in a circular link [46]. Therefore, parenting practices should represent a strategic target of intervention. To better address these issues, further studies could focus on considering various components of parental psychopathology affecting parenting practices and offspring psychopathology outcomes in a longitudinal framework.

## Data Availability

The datasets used and/or analysed during the current study are available from the corresponding author on reasonable request.

## Abbreviations

DSM-IV: Diagnostic and Statistical Manual of mental disorders-IV
CBCL/6–18: Child Behavior Checklist/6–18
ASR: Adult Self Report
IQ: Intellective Quotient
SES: Socioeconomic status
M-Int: Mother Internalizing
M-Ext: Mother-Externalizing
FaLQ: Family Life Questionnaire
DAWBA: Development and Well-Being Assessment
C-Int: Child- Internalizing
C-Ext: Child-Externalizing
SE: Standard Errors
CI: Confidence Intervals
Sd: Standard deviation
Aff.: Affirmation
Disc.: Discipline
Spec. All.: Special Allowances
